# Sleep Disordered Breathing Measures in Early Pregnancy Are Associated with Depressive Symptoms in Late Pregnancy

**DOI:** 10.3390/diagnostics11050858

**Published:** 2021-05-11

**Authors:** Margaret H. Bublitz, Meghan Sharp, Taylor Freeburg, Laura Sanapo, Nicole R. Nugent, Katherine Sharkey, Ghada Bourjeily

**Affiliations:** 1 Department of Psychiatry and Human Behavior, The Warren Alpert Medical School of Brown University, Providence, RI 02903, USA; meghan_sharp@brown.edu (M.S.); Nicole_Nugent@brown.edu (N.R.N.); katherine_sharkey@brown.edu (K.S.); 2 Department of Medicine, The Warren Alpert Medical School of Brown University, Providence, RI 02903, USA; laura_sanapo@brown.edu (L.S.); ghada_bourjeily@brown.edu (G.B.); 3 Women’s Medicine Collaborative, The Miriam Hospital, Providence, RI 02906, USA; taylor_freeburg@brown.edu; 4 The Warren Alpert Medical School of Brown University, Providence, RI 02903, USA; 5 Department of Emergency Medicine, The Warren Alpert Medical School of Brown University, Providence, RI 02903, USA; 6 Rhode Island Hospital, Providence, RI 02905, USA

**Keywords:** pregnancy, perinatal sleep, depression, sleep disordered breathing, obstructive sleep apnea

## Abstract

Sleep disordered breathing (SDB) and depression are both common complications of pregnancy and increase risk for adverse maternal and neonatal outcomes. SDB precedes onset of depression in non-pregnant adults; however, the longitudinal relationship has not been studied in pregnancy. The present research examined temporal associations between SDB and depressive symptoms in 175 pregnant women at risk for SDB (based on frequent snoring and obesity), but without an apnea hypopnea index of ≥5 events per hour at enrollment. Women completed a self-report assessments of depressive symptoms using PHQ-9 and in-home level III sleep apnea monitoring at approximately 12- and 32-weeks’ gestation. We also assessed the risk for SDB using the Berlin Questionnaire in early pregnancy. Results revealed that measures of SDB in early pregnancy as assessed by in-home sleep study, but not by self-reported SDB, predicted elevated depressive symptoms in late pregnancy. SDB in late pregnancy was not associated with depressive symptoms. To conclude, these findings suggest that SDB may increase the risk for elevated depressive symptoms as pregnancy progresses.

## 1. Introduction

Sleep quality worsens during pregnancy [1,2], and poor sleep in pregnancy is associated with adverse outcomes of pre-term birth [3], longer labor and delivery time [4,5], and postpartum depressive symptoms [1]. Sleep disordered breathing (SDB)—a spectrum of disorders characterized by interruptions in airflow due to obstruction of the upper airway that leads to fragmented sleep and diminished sleep quality—can disrupt sleep in expectant mothers. Indeed, the physical changes that accompany pregnancy may predispose women to experience SDB [6], and risk for SDB increases as pregnancy advances. Snoring, a mild form of SDB, increases from 7% of women in the first trimester of pregnancy to up to 48% in the week prior to birth [7]. Obstructive sleep apnea (OSA), a more serious form of SDB, increases from 4–6% of pregnant women in their first trimester to up to 9–20% of women in their third trimester [8,9] and is also associated with adverse maternal health outcomes [10].

While maternal SDB in pregnancy is known to be associated with multiple maternal obstetric complications, such as hypertensive disorders, diabetes, and abnormal fetal growth [9,10,11,12,13], there is limited knowledge about possible associations between SDB in pregnancy and maternal depression. Depression is a common health complication in pregnancy and is associated with adverse maternal, fetal, and child health outcomes [5,14]. A recent meta-analysis identified the prevalence of major depressive disorder as 11.9% during the perinatal period [15].

Previous studies have reported cross-sectional associations between SDB and depressive symptoms in the third trimester [16,17], with recent evidence that the association is also present in early pregnancy [18]. Mellor et al. [19] noted an association between SDB as measured by the Berlin Questionnaire [20] and depressive symptoms between 12 and 39 weeks of pregnancy; however, this association did not withstand adjustment for covariates such as body mass index (BMI). In non-pregnant adults, development of SDB precedes the onset of depression [21], and OSA is associated with higher depression severity. In addition, treatment of OSA with continuous positive airway pressure (CPAP) has been shown to reduce depressive symptom severity [22,23]. Taken together, past research suggests that SDB precedes depression onset in non-pregnant adults, and emerging evidence demonstrates an association between SDB and perinatal depression. However, the longitudinal association between SDB and depression in pregnancy has not yet been elucidated. Understanding the temporal association between SDB and depression across gestation is important because mood and sleep are dynamic processes that change along with the physiologic demands of pregnancy.

Therefore, the aim of this study was to explore the temporal relationship among subjective (self-reported) and objective (in-home sleep study) measures of SDB and self-reported depression across pregnancy. We hypothesized that pregnant women with SDB would be more likely to experience symptoms of depression, and that greater SDB severity would be associated with greater depression severity. As women with SDB symptomatology early in pregnancy would presumably have longer exposure to disturbed sleep throughout the remainder of pregnancy, we also hypothesized that symptoms of SDB in early pregnancy would predict more depressive symptoms in the third trimester.

## 2. Materials & Methods

### 2.1. Recruitment and Participation

For these analyses we examined a convenience sample of pregnant participants at high risk for SDB who were enrolled in a study assessing predictors of risk for OSA. We approached potential participants at prenatal care appointments and queried them about snoring. Inclusion criteria were self-reported habitual snoring (defined as 3–4 times/week), AHI < 5 at in-home sleep testing at 12–14 weeks’ gestation, BMI ≥ 27 kg/m^2^ at recruitment, <18 weeks’ gestation at enrollment, ≥18 years of age, pregnant with a singleton pregnancy, and ability to provide informed consent. Women were excluded if they had a history of OSA treated with CPAP prior to or during pregnancy given past evidence that CPAP therapy may reduce symptoms of depression [22,23]. We did not exclude women with a previous history of depression. All participants completed an in-home sleep study at approximately 12 weeks’ gestation to measure apneas and hypopneas during sleep, and self-reported SDB symptoms using the Berlin Questionnaire [20]. At approximately 14 weeks’ gestation they completed self-report measures of depressive symptoms. The in-home sleep study and depression measure were repeated at approximately 32 weeks’ gestation. All subjects gave their informed consent for inclusion before they participated in the study. The study was conducted in accordance with the Declaration of Helsinki, and the protocol was approved by the Ethics Committee of Rhode Island Hospital (#781944).

### 2.2. Measures

In-home sleep testing. In-home sleep studies were performed using the Nox T3 device (Carefusion, San Diego, CA, USA). This device uses built-in sensors to record nasal pressure and snoring as well as body position and activity measured by a 3D built-in accelerometer sensor, and electrocardiography. Respiratory effort is measured with dual abdominal/thoracic respiratory inductance plethysmography belts. Continuous oxygen saturation is measured with a wireless Bluetooth pulse oximeter. The Nox T3 auto-score algorithm has been validated with in-laboratory polysomnography (in non-pregnant adults) and the auto-score apnea hypopnea index (AHI) strongly correlates with AHI derived from polysomnography (r = 0.93) [24]. All studies were scored by a certified polysomnographic technologist supervised by the investigative team. Apneas were defined as a drop in peak signal excursion by >90% of pre-event baseline and lasting for more than 10 s. Hypopneas were defined using the recommended (3%) desaturation rule by the American Academy of Sleep Medicine [25]. Sleep disordered breathing (if developed by 32 weeks’ gestation) was defined as an apnea hypopnea index of five events or more per hour.

Berlin Questionnaire. Participants completed the Berlin Questionnaire at approximately 12 weeks’ gestation. The Berlin Questionnaire is a 10-item self-report measure that asks participants to report on snoring (category 1), daytime somnolence (category 2), and hypertension and BMI (category 3). Scores on categories 1 or 2 are considered positive if responses indicate frequent snoring or somnolence (>3–4 times/week), and scores on the 3rd category are considered positive if participants report a history of hypertension or BMI of >30 kg/m^2^. A positive score on 2 or more categories indicated high risk for OSA [20]. Given that inclusion criteria for the study required women to have a BMI ≥ 27 kg/m^2^ at recruitment, we also calculated Berlin scores using only categories 1 and 2 to prevent artificially inflating the number of positive responses.

Depressive Symptoms. Symptoms of prenatal depression were measured using the Patient Health Questionnaire (PHQ)-9 [26]. The PHQ-9 is a self-report measure of depressive symptoms in the previous two weeks. Items correspond to each of the nine diagnostic symptoms of major depressive disorder as defined by the diagnostic and statistical manual of mental disorders (DSM). Responses range from 0 (not at all) to 3 (nearly every day). Scores of 10 or greater reflect clinically significant levels of depressive symptoms. To adjust for the confounding influence of disrupted sleep as both a symptom of depression and OSA, we also calculated a depression score omitting the sleep item, as has been done in previous research [17].

### 2.3. Statistical Analyses

We performed descriptive statistics to summarize participant demographic characteristics. AHI values were significantly skewed (skewness > 2) and log transformed prior to analyses. To test our study hypotheses, we performed linear regression analyses to examine (1) cross-sectional associations among AHI and Berlin Questionnaire and depressive symptoms within early (12–14 weeks’ gestation) and late (32 weeks’ gestation) pregnancy, and (2) longitudinal associations between AHI and Berlin Questionnaire in early pregnancy and depressive symptoms (PHQ-9 score) in late pregnancy (adjusting for depressive symptoms in early pregnancy). Covariates included maternal age, BMI assessed at enrollment, race, ethnicity, daytime sleepiness in early pregnancy (measured by the Epworth Sleepiness Scale [27]), self-reported history of a depression diagnosis, and self-reported antidepressant medication use in pregnancy. We also performed sensitivity analyses by repeating linear regression analyses after excluding women who reported taking antidepressant medications in pregnancy. IBM SPSS Statistics 25 was used for statistical analyses.

## 3. Results

Demographic characteristics. 175 women were included in this study. Women were, on average, 29 years old (range: 18–44), 12 weeks’ gestation at enrollment (SD = 7 weeks), BMI measured at the first study visit was 34 kg/m^2^ (SD = 7, range: 27–55 kg/m^2^), and 27.8% of the sample was primiparous. Self-identified ethnic and racial distribution was as follows: 32% Latina, 56% White, 20% Black, 3% Asian, 4% American Indian/Alaskan Native, 10% multiracial, and 7% listed their race as “unknown”. A total of 5.2% of women reported antidepressant use in pregnancy, and 33% reported a diagnosis of depression prior to pregnancy. As expected, given the inclusion criteria, all women reported snoring at least 3 times per week at enrollment, and 77% had a score of 2 or more on the Berlin Questionnaire in early pregnancy, indicating high risk for OSA. Exactly 50% had a positive score on the Berlin Questionnaire when category 3 was omitted from scoring. Mean AHI at enrollment was 0.99 (SD = 1.12, range: 0–4.70). A total of 126 participants repeated the in-home sleep study at 32 weeks’ gestation. Mean AHI at 32 weeks’ gestation was 4.81 (SD = 5.43, range: 0–47.20). Among these women, 40% (N = 50) had an AHI > 5 using the Nox 3 scoring algorithm. Furthermore, 19% of women reported clinically elevated symptoms of depression (PHQ-9 > 9) in early pregnancy, and 20% reported clinically elevated symptoms of depression in late pregnancy. See Table 1.

**Figure 1:**
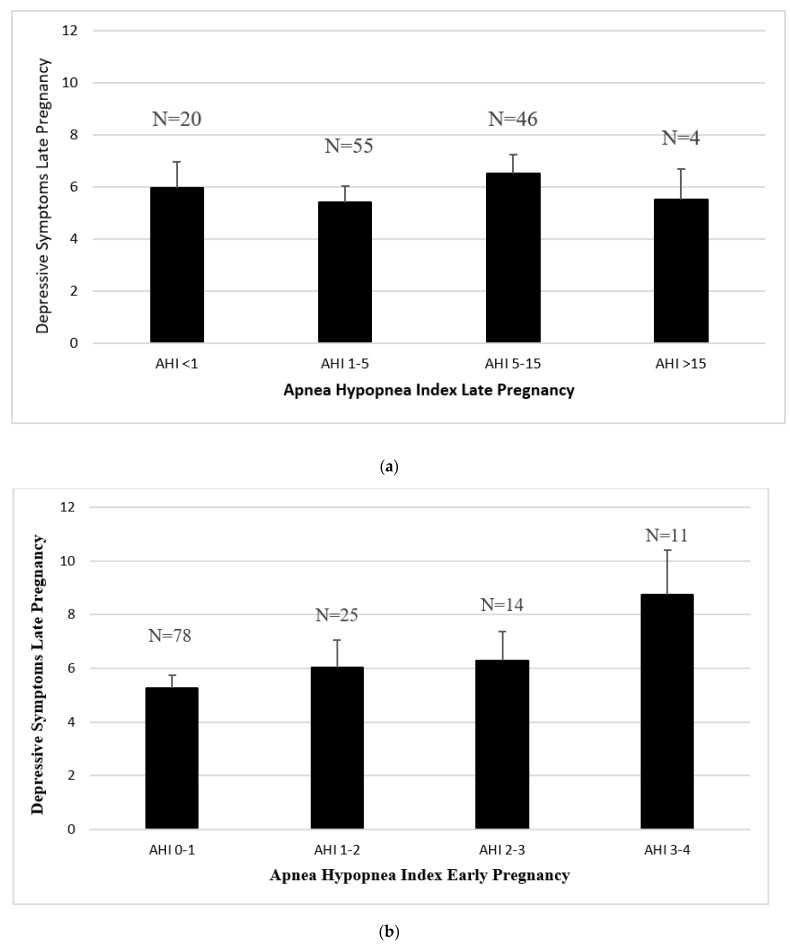
(**a**) Apnea Hypopnea Index in late pregnancy and depressive symptoms in late pregnancy. Note. Apnea hypopnea index was measure by in-home sleep study. Depressive symptoms were measured using the Patient Health Questionnaire-9. Analyses adjusted for maternal age, BMI assessed at enrollment, baseline depressive symptoms, race, ethnicity, daytime sleepiness, history of a depression diagnosis, and antidepressant medication. AHI: Apnea Hypopnea Index. (**b**) Apnea Hypopnea Index in early pregnancy and depressive symptoms in late pregnancy. Note. Apnea hypopnea index was measure by in-home sleep study. Depressive symptoms were measured using the Patient Health Questionnaire-9. Analyses adjusted for maternal age, BMI assessed at enrollment, baseline depressive symptoms, race, ethnicity, daytime sleepiness, history of a depression diagnosis, and antidepressant medication. AHI: Apnea Hypopnea Index.

Sleep Disordered Breathing and Depressive Symptoms. Cross-sectional analyses did not demonstrate significant associations between objective measures of SDB and symptoms of depression. Specifically, we did not observe significant cross-sectional associations among AHI and depressive symptoms in early pregnancy (B = 0.11, *p* = 0.16) or late pregnancy (B = −0.01, *p* = 0.89, See Figure 1a). We did not observe a significant association between elevated scores on the Berlin Questionnaire and depressive symptoms in early pregnancy (F = 0.74, *p* = 0.39). However, when we omitted category 3 from Berlin scoring, we noted a positive association between the Berlin Questionnaire and depressive symptoms in early pregnancy (F = 8.93, *p* = 0.003), and this remained significant when we removed the sleep item from the PHQ9 (F = 7.72, *p* = 0.006).

Analyses did support a significant longitudinal association between AHI scores in early pregnancy and elevated depression scores in later pregnancy. After adjusting for covariates, higher AHI in early pregnancy was significantly associated with more depressive symptoms in late pregnancy (B = 0.22, *p* = 0.012). The association remained significant after excluding the sleep item from the PHQ-9 score (B = 0.25, *p* = 0.004). The association also remained significant after excluding women who reported antidepressant use in pregnancy (B = 0.19, *p* = 0.043). See Table 2, Figure 1b. We did not observe a significant association among elevated scores on the Berlin Questionnaire in early pregnancy and depressive symptoms in late pregnancy (F = 0.43, *p* = 0.51). Results did not change when we omitted category 3 from Berlin scoring (F = 0.08, *p* = 0.78).

## 4. Discussion

In this study, we showed that objective measures of SDB in early pregnancy predicted depressive symptoms in late pregnancy. Moreover, this association remained significant while adjusting for baseline depression symptoms and antidepressant use in our statistical model. One implication of these results is that, during early pregnancy, the presence of apneas and hypopneas that do not meet standard criteria for OSA (i.e., AHI > 5) may in fact confer an important risk to maternal health later in pregnancy. Moreover, it suggests that, although their mood symptoms were recognized and treatment with antidepressant medication was attempted, an important contributor to those symptoms—namely SDB and resulting sleep disturbance—may have been overlooked or assumed to be non-contributory given that these measures fell below the clinically defined cut-off early in pregnancy.

Due to the parent study design, women with a history of OSA treated with CPAP prior to or during pregnancy were excluded from participating and did not have follow up depression evaluation in late pregnancy. Nonetheless, as all women reported loud snoring in pregnancy, and given that we and others have argued that the proper objective definition of SDB in pregnancy has not been clearly determined [28,29], we decided to examine the association of measures of SDB in early pregnancy that fell below the conventionally defined cut-off (i.e., women with AHI values < 5 on sleep study) with depressive symptoms. The observed association between AHI in early pregnancy and depressive symptoms in late pregnancy emphasize the potential low utility of a cut-off for AHI of 5 events per hour, as an association with pathology is observed below that cut-off. AHI values below the conventional cut-off may still represent a significant exposure to SDB pathology that increases vulnerability for depressive symptoms in later pregnancy. In addition, rates of OSA by late pregnancy approached 40% in this sample, indicating that AHI > 5 is an imprecise marker of SDB pathophysiology, particularly in early pregnancy.

Although depression and OSA have been extensively studied among non-pregnant populations [30,31], few studies have evaluated this association in pregnancy. Mellor et al. reported that sleep disturbances and depressive symptoms were associated in pregnancy; however, the relationship was attenuated when accounting for other depression risk factors such as BMI [19]. Our results partially replicated the findings from the Mellor study. Mellor et al. evaluated SDB using the Berlin Questionnaire and did not include an objective measure of SDB such as an in-home sleep study. They found that positive scores on the Berlin Questionnaire were associated with elevated depressive symptoms; however, this did not withstand adjustment for BMI. As well, the Mellor study had only a small percentage of women with obesity, and a smaller proportion of women with a history of depression. In the current study, after omitting category 3 from the Berlin Questionnaire scoring which may have been artificially inflated due to inclusion criteria of the parent study that women have BMI ≥ 27 kg/m^2^ at recruitment, participants considered high-risk for OSA based on reported snoring and somnolence also reported elevated depressive symptoms in early pregnancy, but not late pregnancy. These findings suggest that symptoms of frequent snoring and somnolence are associated with depressive symptoms in early pregnancy, but SDB symptoms may not exacerbate risk for worsening depressive symptoms over gestation. Notably, questionnaires have yet to be validated for SDB screening in pregnancy [32], and most women in our sample (77%) were categorized as high-risk for OSA on the Berlin Questionnaire in early pregnancy despite having AHI values below the diagnostic criteria for OSA. In addition, a recent study that also evaluated the Berlin Questionnaire by category reported that positive scores on category 3 (BMI and high blood pressure) predicted adverse obstetric outcomes whereas scores on the other categories did not [33]. The authors concluded that the Berlin Questionnaire, when used in pregnancy, is likely reflecting excessive maternal weight rather than OSA. Hence, screening for OSA using the Berlin Questionnaire alone would not be recommended, particularly in early pregnancy and among women with elevated BMI. This recommendation is aligned with results by Tantrakul et al. who found low predictive value of the Berlin questionnaire in first trimester when compared to objectively measured OSA [34]. In addition, obesity is a risk factor for the development of both depression and SDB [35,36]. In our sample, 33% of women reported a history of depression, and rates of elevated maternal depressive symptoms ranged from 19–20%. These rates are higher than those reported in national samples [15,37]. Therefore, this sample may represent a particularly vulnerable group for the development of maternal depression.

The association between SDB and depression is well established [30,31], yet the exact mechanisms underlying the relationship have yet to be determined. Some prior studies have suggested that inflammatory pathways may mediate the relationship, since both OSA and depression are associated with the release of pro-inflammatory cytokines [38,39,40]. Others have suggested that intermittent hypoxia, a symptom of OSA, may lead to the development of cerebral small vessel disease and blood-brain barrier dysfunction and thus result in the development of depressive symptoms [41]. More research is needed to understand the biological underpinnings of these associations.

The association between SDB and depression in pregnancy is important to consider in clinical care. Early identification of risk factors for depression can assist in monitoring and management of mood disorders and offers opportunities for non-pharmacological interventions. In studies conducted in the general population, CPAP was found to reduce depression and anxiety in patients with severe OSA after several months of treatment [41]. However, few studies have evaluated the impact of CPAP in pregnancy [42], particularly as it relates to perinatal mood. In addition, barriers to CPAP treatment in pregnancy include risk for poor adherence, which may be secondary to baseline poor sleep [3] or demands of childcare and other responsibilities that may take precedence over their health. Future research on the effects of early CPAP usage on prenatal depressive symptoms is warranted given the findings in this study and the challenges associated with treating SDB in pregnancy.

Strengths of this study include the objective measurement of OSA with in-home sleep studies, repeated measures of depression in early and late pregnancy, and the racial and ethnic diversity in our sample. Results should be interpreted in light of several limitations. Most notably, participants were selected to be at risk for the development of SDB based on obesity status and habitual snoring, but with AHI scores < 5 events per hour in early pregnancy. Therefore, our results may not be generalizable to pregnant patients at lower risk for SDB, or those with more severe disease, due to sample size. In addition, while rates of SDB increased substantially across gestation in this sample, rates of depression remained relatively stable. Future studies are needed that evaluate the temporal association between SDB and depression among women at higher risk for prenatal depression. We did not measure postpartum depressive symptoms in this study; future research should be conducted to understand the relationship between OSA and depression in the postpartum period. Lastly, due to the COVID-19 pandemic, a number of participants who were expected to have follow-up 3rd trimester visits were unable to do so, limiting the number of participants with longitudinal data.

## 5. Conclusions

To conclude, measures of SDB in early pregnancy that fall below the conventional cut-off for objectively determined SDB predicted depressive symptoms in late pregnancy. These findings suggest that SDB may increase the risk for elevated prenatal depressive symptoms as pregnancy progresses. Screening for SDB early in pregnancy among women with established risk factors may therefore be beneficial to reduce both SDB-associated adverse obstetric and psychiatric outcomes. More research is needed to determine if treatment of sleep disordered breathing (e.g., CPAP) could reduce the risk for depressive symptoms later in pregnancy.

## Figures and Tables

**Table 1:** Demographic characteristics.

	Mean (SD)/%
Maternal Age (years)	29 (6)
Primiparous	27.8%
Race (%)	
White	56%
Black	20%
Asian	3%
Native American/Alaskan Native	4%
Multiracial	10%
Ethnicity (% Hispanic)	32%
Prenatal Body Mass Index kg/m^2^	34 (7)
Self-reported History of Depression	33%
Self-reported use of Antidepressant Medication	5.2%
Epworth Sleepiness Scale (early)	9.58 (4.80)
Epworth Sleepiness Scale (late)	7.75 (3.69)

**Table 2:** Linear regression models of Apnea Hypopnea Index in early pregnancy predicting depressive symptoms in late pregnancy.

Model	Depression Scale	β	SE	R^2^	*p*-Value
1	PHQ9	0.20	1.89	0.04	0.026
2	PHQ9 + Covariates	0.22	1.89	0.20	0.012
3	PHQ9 without sleep item + Covariates	0.25	1.60	0.23	0.004
4	PHQ9 + Covariates, no antidepressants	0.19	1.99	0.19	0.043

Note. AHI values were significantly skewed, therefore log transformed values were included in analyses. Covariates included: maternal age, BMI assessed in pregnancy, race, ethnicity, daytime sleepiness (measured by the Epworth Sleepiness Scale in early pregnancy), history of a self-reported depression diagnosis, and self-reported antidepressant medication use in pregnancy (with the exception of Model 4). PHQ9: Patient Health Questionnaire.

## Data Availability

The data presented in this study are available on request from the corresponding author.

## Abbreviations

AHI	apnea hypopnea index
BMI	body mass index
CPAP	continuous positive airway pressure
DSM	diagnostic and statistical manual of mental disorders
OSA	Obstructive sleep apnea
PHQ-9	Patient Health Questionnaire
SDB	Sleep disordered breathing
